# The Multiple Facets of Lutein: A Call for Further Investigation in the Perinatal Period

**DOI:** 10.1155/2016/5381540

**Published:** 2016-09-07

**Authors:** Serafina Perrone, Monica Tei, Mariangela Longini, Giuseppe Buonocore

**Affiliations:** ^1^Pediatric Unit, Department of Molecular and Developmental Medicine, University of Siena, Siena, Italy; ^2^UOC Clinical Pathology, AOUS, Siena, Italy

## Abstract

Lutein may have important antioxidant actions in free-radical-mediated diseases, in addition to its well-known antioxidant and cytoprotective effects on macula and photoreceptors. The peculiar perinatal susceptibility to oxidative stress indicates that prophylactic use of antioxidants as lutein could help to prevent or at least to reduce oxidative stress related diseases in newborns. Since lutein is not synthesized by humans, the intake primarily depends on diet or supplementation. Newborns receive lutein exclusively from breast milk. Lutein supplementation in term newborns has been reported to reduce oxidative stress and increase antioxidant capacities in the first days of life. Innovative frontiers concerning lutein supplementation are orientated toward cardiometabolic health improvement and cognitive benefits. The safety of lutein as an antioxidant agent has been confirmed in experimental and clinical studies, but its routine use is not recommended in perinatal period. This review summarizes what is known about the role of lutein as an antioxidant and anti-inflammatory agent in animal model and humans.

## 1. Structure and Location of Lutein

Lutein is a fat-soluble pigment, belonging to the family of carotenoids, which encompasses about 700 members in nature. Carotenoids are divided into two classes according to their chemical structure: the carotenes (hydrocarbons, such as *β*-carotene and lycopene) and the xanthophylls (polar compounds including oxygen atoms in their structure, such as lutein and its structural isomer zeaxanthin) [1]. Since xanthophyll biosynthesis occurs exclusively in plants, algae, bacteria, and certain fungi [2], the primary intake of lutein depends on diet or supplementation. Lutein and zeaxanthin can be found in yellow-orange food, such as egg yolk and corn [3], but especially in dark green vegetables such as turnip greens, kale, parsley, spinach, and broccoli [4]. Lutein intake from dietary sources is strongly associated with plasma concentrations [5]. Indeed, it has been shown that in humans every 10% increase in dietary lutein corresponds to a 2,4% increase in serum lutein concentration [6]. In human body lutein is stored in the eye (retina, rod outer segments, and lens) [7, 8] and other places in human body including skin [9], cervix, brain, and breasts.

The chemical structure of lutein (C_40_H_56_O_2_) consists of 40-carbon, hence known as tetraterpenoids, with alternating single and double carbon-carbon bonds with attached methyl side groups. The presence of a hydroxyl group at both ends of the molecule distinguishes lutein and zeaxanthin from other carotenoids and it is responsible for the high chemical reactivity with singlet oxygen [10–12]. The presence of electrons localized over the entire length of the hydrocarbon chain molecules allows the neutralization of free radicals (FR) [13]. Due to its modest aqueous solubility, lutein is usually localized in the inner core of the cell membranes or bound to proteins [14]. Since cell membranes are the first structures attacked by FR, the anchor of lutein guarantees protection. Lutein also crosses the blood-brain barrier and the placenta; its presence is three times higher in breast milk and colostrum, compared to those of other carotenoids, as a result of an active secretion from the bloodstream. The plasma levels of lutein in the mother correlate with carotenoid status in the newborn [15]. In the neonatal period, fresh human milk is the main source of lutein [16]. Mature human milk can be stored safely in a freezer and heated in a microwave oven without loss of carotenoids [17]. Lutein-enriched infant formulas are now available. Oral supplementation represents an alternative source that has been demonstrated to decrease oxidative stress (OS) biomarkers and increase biological antioxidant potential in the first days of human life [18, 19].

## 2. Antioxidant, Anti-Inflammatory, and Neuroprotective Properties

Several antioxidant activities have been ascribed to lutein: inhibition of membrane lipids peroxidation, particularly in photoreceptors, which have plenty of polyunsaturated fatty acids; direct antioxidant action; and anti-inflammatory and immunomodulatory properties.

**Figure 1:**
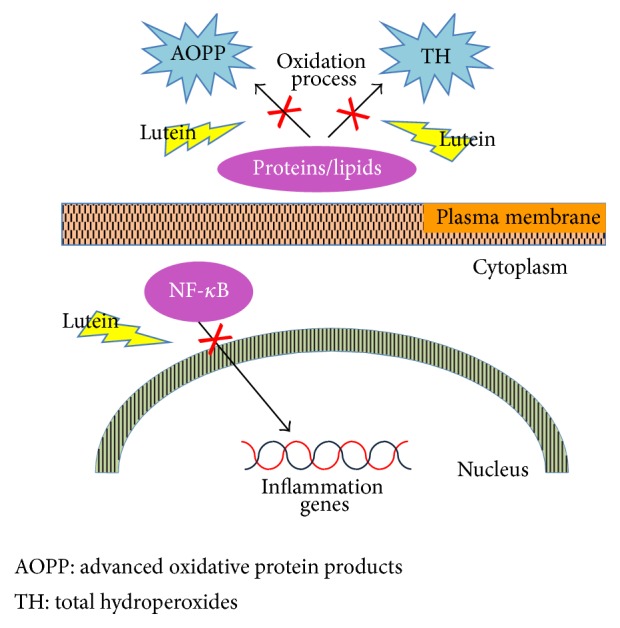
Schematic representation of anti-inflammatory and antioxidant effects of lutein.

In a rat model of endotoxin-induced uveitis lutein blocks the degradation of inhibitory kB-a from the cytosolic fraction and prevents NF-kappa-B (NF-*κ*B) translocation, decreasing inducible gene transcription and synthesis of inflammatory mediators (Figure 1) [20, 21].

Paraquat and hydrogen peroxide-induced apoptosis are neutralized by lutein in cultured retina photoreceptors promoting survival and differentiation [22]. Lutein also avoids the photooxidation of phosphatidyl-pyridinium bisretinoid (A2-PE), which may activate a cascade of events leading to the formation of reactive species in retinal pigment epithelial cells [23]. Moreover, lutein supplementation in retinal pigment epithelial cells prevents the proteasome inactivation in response to photooxidation and modulates inflammation-related genes [24].

In lipopolysaccharide- (LPS-) stimulated macrophages line, lutein has been found to decrease intracellular hydrogen peroxide (H_2_O_2_) accumulation by scavenging superoxide anion and H_2_O_2_ [25]. In the same study, lutein has been found to inhibit the expression of proinflammatory genes by suppressing nuclear factor NF-*κ*B translocation and reducing LPS-induced secretion of tumor necrosis factor- (TNF-) *α* and interleukin-1*β*. Similar anti-inflammatory mechanisms have been observed* in vitro* in both models of gastric epithelial cells [26] and microglia [27]. Lutein also significantly reduces skin inflammatory responses in ultraviolet-irradiated keratinocytes [28].

Moreover, lutein acts as a competitive inhibitor of cytosolic calcium-dependent phospholipase A_2_ inhibiting arachidonic acid release from a macrophage cell line [29]. In vascular smooth muscle cells, platelet-derived growth factor and extracellular H_2_O_2_ stimulation induce FR production, which is attenuated by lutein [30].

The protective effects of lutein against protein oxidation, lipid peroxidation, and DNA damage induced by OS have been reported also in human lens epithelial cells where lutein supplementation increased reduced glutathione (GSH) levels and reduced/oxidized GSH ratio [31].

Supplementation with lutein has anti-inflammatory, neuroprotective, and antiangiogenic properties. In mice receiving three-month lutein supplementation, the outer nuclear layer thickness histopathologically examined was significantly greater than in the nonsupplemented group. In the same cohort, retinal expression of proinflammatory mediators such as inducible nitric oxide synthase, TNF-*α*, cyclooxygenase-2, IL-1*β*, and vascular endothelial growth factor was significantly lower in supplemented mice [32].

The administration of lutein affords neuroprotective effect against transient cerebral ischemic injury in mice since it is able to significantly increase reduced/oxidized GSH ratio as well as activities of antioxidant enzymes (superoxide dismutase, GSH peroxidase, and catalase) [33].

Lutein suppresses STAT3 activation by inflammatory cytokines and extracellular signal-regulated kinase activation, slowing DNA damage and preserving a-wave electroretinogram amplitude in mouse models [34]. Lutein plays a neuroprotective role in retinal ganglion cells against N-methyl-D-aspartate-induced retinal damage in rats [35].

Lastly, lutein treatment significantly decreased OS in rat model of skeletal ischemia/reperfusion injury by downregulating oxidative stress and inflammatory mechanisms [36].

## 3. Lutein and Cognitive Function

Recent papers report how lutein, predominantly accumulating in the brain, is positively associated with improved cognitive function in the elderly [37]. Macular pigment optical density, which is a stable measure of lutein and zeaxanthin in the retina, is consistent with better global cognition, verbal learning and fluency, and processing and perceptual speed in old people [38–40]. Moreover, lutein improves cognitive scores after 4-month supplementation in old women [41] and ameliorates visual processing speed and visual motor behavior in young subjects [42].

Due to the encouraging findings of a positive impact of lutein on brain function, growing interest focuses on identifying possible lutein functions in neurodegenerative diseases such as Parkinson disease (PD) and Alzheimer disease (AD). It has been suggested that lutein offers benefits against neuronal damage occurring in AD by virtue of its mitochondrial protective, antioxidant, and antiapoptotic properties. In a randomized, double-blind clinical trial, AD patients were daily supplemented for six months with macular carotenoids (10 mg meso-zeaxanthin, 10 mg lutein, and 2 mg zeaxanthin) [43]. The authors found significant improvements in visual function and increase of macular pigment density in patients with AD after lutein supplementation while cognitive function was not influenced. In PD-mice model, lutein has been found to protect nigral dopaminergic neurons by enhancing antioxidant defense mechanisms and diminishing mitochondrial dysfunction and apoptotic death [44]. Lutein reversed the loss of nigral dopaminergic neurons by inhibiting the activation of proapoptotic markers (Bax and caspases 3, 8, and 9) and enhancing antiapoptotic marker (Bcl-2) expressions, with significant reduction in motor abnormalities. These findings pave the way to a beneficial employment of lutein for neurodegenerative therapy even if its potential protective function against these diseases remains to be explored.

## 4. Lutein and the Eye

In human eye macular pigment is composed of three carotenoids including lutein in equal concentrations to zeaxanthin and meso-zeaxanthin [45–47]. The* macula lutea* is a yellow, circular area 5-6 mm in diameter, located in the central and posterior portion of the primate retina. The macula includes the majority of photoreceptors and it is responsible for central vision and high-resolution visual acuity. Neuronal lipid bilayer membranes in the retina are especially vulnerable to oxidative damage because of exposure to high oxygen concentration. Since lutein is soluble in polyunsaturated phospholipid membrane domains, it plays a pivotal role against OS in retinal tissues. Retinal vulnerability to hypoxia-ischemia is evident especially as a result of photochemical damage, primarily located in the outer layers of the central region of the retina, regarding both photoreceptors and retinal pigment epithelium [48]. Laboratory studies have suggested that photochemical damage is triggered by oxidative events leading to retinal cells apoptosis [49]. In particular, ocular exposure to sunlight, UV, and short blue light-emitting lamps may lead to cataract and retinal degeneration through a photooxidation reaction. In photooxidation reactions, phototoxic chromophores in the eye are able to absorb light but they subsequently turn to an unstable state (singlet and then a triplet state) producing FR [49]. Antioxidant quenchers as lutein can prevent the phototoxic reactions damage. In fact, due to its chemical structures with extensive conjugated bonds, lutein is able to absorb light of the blue range wavelength (400–500 nm) preventing light-induced retinal damage [50, 51]. Moreover, lutein acts as an effective quencher of singlet molecular oxygen (^1^O_2_) in the retina during OS conditions, preventing lipid peroxidation and the accumulation of FR responsible for photoreceptor apoptosis [11, 12, 52]. OS also occurs in the inner part of the retina, particularly within axons of retinal ganglion cells which are rich in mitochondria and consequently sensitive to FR harmful effects compared to neuron soma [53].

OS is the main consequence of retinal ischemia which was found to underlie diabetic retinopathy (DR) and retinopathy of prematurity (ROP) [54]. In both DR and ROP early ischemia due to abnormal retinal blood supply leads to abnormal neovascularization and subsequent hemorrhages and blindness. In preterm babies the hypoxic injury is caused by an imbalance between an increased metabolic demand and delayed retinal vascular development due to the suppression of growth factor in a hyperoxic environment [55]. DR hyperglycemia and decrease in blood flow produce retinal ischemia [56]. Hyperglycemia induces several changes including leukostasis, vasoconstriction, and a proinflammatory state that also cause hypoxia in the retina. The early proinflammatory changes can directly provoke hypoxia in the retina.

Furthermore, lutein is well known to be protective against senile cataract by influencing changes in glutathione oxidation, which is responsible for the increased susceptibility of the nucleus to oxidative damage in older lenses [57]. Protective effects of lutein have been also demonstrated in age-related macular degeneration (AMD). AMD is a major cause of visual impairment and blindness among people 65 years or older. It is due to the decrease in naturally protective antioxidant systems and the increase in UV and visible light-absorbing endogenous phototoxic chromophores that produce reactive oxygen species [58]. Lutein counteracts stress-induced changes in the retinal pigment epithelium promoting tight junction repair and suppresses inflammation both by direct scavenging and by induction of endogenous antioxidant enzymes.

Sustained supplementation of lutein, zeaxanthin, and meso-zeaxanthin was demonstrated to be effective in increasing macular pigment, contrast sensitivity, and visual function in early AMD [59]. These three carotenoids showed also beneficial effects on visual performance in various retinal diseases [60].

In a large multicenter double-masked clinical trial called Age-Related Eye Disease Study 2 (AREDS2), participants were randomly assigned to receive four different treatments: (1) 10 mg lutein + 2 mg zeaxanthin; (2) fish oil containing eicosapentaenoic acid (EPA) and docosahexaenoic acid (DHA); (3) lutein + zeaxanthin + DHA + EPA; and (4) placebo. Lutein + zeaxanthin formulation significantly decreased the progression to advanced AMD [61].

Interestingly, lutein, meso-zeaxanthin, and zeaxanthin supplementation has been reported to be effective in ameliorating contrast sensitivity in healthy population (free of retinal disease) by increasing retinal concentrations of these carotenoids [62]. A recent meta-analysis by Ma et al. reports that lutein, zeaxanthin, and meso-zeaxanthin supplementation improves macular pigment optical density in both AMD and healthy subjects with a dose-response relationship [63].

## 5. Lutein and Cardiometabolic Health

Due to its antioxidant and anti-inflammatory capacity, lutein has been shown to exert a positive influence in promoting cardiovascular health and decreasing the risk of Coronary Artery Disease (CAD). Animal studies show that lutein contributes to prevention of atherosclerosis development by decreasing malondialdehyde and oxidized low-density lipoprotein levels and reducing inflammatory cytokines such as interleukin- (IL-) 10 [64]. Furthermore, in ApoE-deficient mice supplemented with lutein for 24 weeks NADPH oxidase was inhibited and peroxisome proliferator-activated receptor expression was increased by lutein, protecting against high fat diet-induced atherosclerosis [65].

The possible beneficial cardiovascular effect of a lutein-rich diet in humans, particularly in preventing arterial plaque formation, has been reported in the Atherosclerosis Risk in Communities (ARIC) and the Carotid Ultrasound Disease Assessment (CUDAS) studies [66, 67]. An inverse association between plasmatic lutein and atherosclerosis is also shown in the Los Angeles Atherosclerosis Study [68]. While the benefits regarding hypertension are uncertain [69], lutein has been reported to counteract OS produced after myocardial ischemia/reperfusion damage [70, 71]. Upon reperfusion, neutrophils accumulate and produce an inflammatory response with increased generation of highly reactive oxygen species, which are responsible for myocytes apoptosis [70]. Consequently, limiting myocardial injury may prevent contractile dysfunction, reducing morbidity and mortality associated with CAD [72].

A recent meta-analysis also showed a lower risk of coronary heart disease, stroke, and metabolic syndrome in high-lutein blood concentration subjects or lutein-supplemented subjects [73].

## 6. Lutein and Oxidative Stress in Perinatal Period

Oxidative stress is defined as an imbalance between free radicals, such as nitric oxide (NO^•^), superoxide anion (O_2_^•−^), and H_2_O_2_, and antioxidants, promoting overabundance of FR. The newborn is particularly susceptible to OS due to the sudden transition from uterine life, relatively hypoxic, to extrauterine environment, with significantly higher oxygen concentrations. Other predisposing factors are the rapid tissue growth and perinatal conditions characterized by increased concentrations of FR and free iron, such as chorioamnionitis, placental hypoperfusion, neonatal hypoxic-ischemic events, inflammation, or fetal-placental transfusion.

Several preterm newborn's diseases, such as retinopathy of prematurity (ROP), bronchopulmonary dysplasia, intraventricular hemorrhage, periventricular leukomalacia, necrotizing enterocolitis, oxidative hemolysis, and renal failure [74–78], recognize in OS a pathogenetic role. These pathological conditions were grouped into a larger entity defined as “FR disease of the newborn” [79].

Therefore one of the goals of modern neonatology is to protect the infant from oxidative damage by reducing the production of FR or promoting the development of antioxidant systems. Vitamins, FR inhibitors, and scavengers have been used as antioxidant drugs in clinical and experimental studies with uncertain results. Among them, lutein represents one of the antioxidant strategies with clinical application in the perinatal period [80]. Newborns receive lutein from breast milk: lutein is the predominant carotenoid in mature breast milk [81, 82]. Breast-feeding infants intake of lutein depends on multiple factors such as maternal lutein intake, alcohol consuming, smoking [83], and maternal body mass index; for example, breast milk of obese mothers was found to have lower lutein content [84]. A recent paper by Vishwanathan et al. shows that lutein is the prevalent carotenoid in the developing infant brain and its concentration is lower in preterms compared to term neonates perhaps for lack of supplementation [85].

Few data are currently available about the effects of lutein supplementation in newborns. Lutein may play a role in visual development, being involved in cell maturation in the developing macula [86]. Moreover, a recent clinical trial showed that lutein supplementation may improve neuroretinal health (assessed through electroretinography recording the voltage change across the retina after light stimulus) in preterm newborn infants [87]. Although oral lutein is well absorbed by preterm babies [88], it has not yet been verified whether dietary lutein enhances visual development in infants [89] and the mechanisms remain largely not understood.

Since ROP is OS-related disease, a striking interest has been focused on the possible role of lutein in preventing it [90, 91]. ROP is a two-phase disease affecting preterm infants. At first, the hyperoxic stimulus during oxygen supplementation downregulates the vascular endothelial growth factor with subsequent interruption of retinal vessel growth. Afterwards, the condition of relative hypoxia of the retina, occurring when the babies stop oxygen therapy, leads to the abnormal proliferation of vessels (neovascularization) and consequently OS [92].

Data regarding the possible benefits of lutein supplementation in preventing ROP are scarce and no consensus has been achieved yet. In a multicenter, randomized-controlled trial, the incidence of ROP in very low birth weight infants was found not to decrease after lutein supplementation. Similar findings were described also in another randomized-controlled trial by Dani et al. [93]. Romagnoli et al. showed a strong antioxidant capacity of lutein, which significantly increased the biological antioxidant potential but not efficacy to reduce the occurrence/severity of ROP [94]. Although a significant linear correlation was reported between plasma lutein concentration and total antioxidant status, supplementation with lutein orally was ineffective in enhancing biological antioxidant capacity in preterm babies [95]. Conversely, clinical trials in term healthy newborns indicated that orally supplemented lutein was effective in enhancing biological antioxidant potential and reducing lipid peroxidation [18, 19]. As demonstrated so far, lutein has a well-ascertained antioxidant and anti-inflammatory role, while the capacity preventing OS-related newborn diseases remains uncertain, probably due to the multifactorial nature of the pathological processes or the need for higher daily doses.

Further clinical trials are needed to evaluate therapeutic effects of lutein on preterm and term infant morbidity, particularly the free-radical-mediated diseases of the newborn.

## 7. Conclusions

Due to its antioxidant anti-inflammatory properties and safety, lutein has been considered as a promising molecule in several fields of application. Neonatal age is a vulnerable period regarding the threatening effects of OS on the developing tissues. Neonates, especially if preterm, are defenseless against the oxidative cellular injury because of both several prooxidant events, such as the exposition to a relatively hyperoxic environment with enhanced generation of FR, and deficient antioxidant systems. Additional neonatal conditions (inflammation, hypoxia, ischemia, and free iron release) may also worsen OS damage. As a consequence, a great deal of interest has been focused on antioxidant treatments. The efficacy of lutein in counteracting oxidative damage has been tested in human adult diseases, such as atherosclerosis, AMD, and senile cataract. This evidence calls for a further investigation in infants. Since humans do not synthesize lutein, lutein supplementation should be undertaken in maternal diet and in all non-lutein-enriched formula fed newborns, lacking an adequate dietary intake.
